# A clinical study on arthroplasty for failed internal fixation of hip fractures and review of literature

**DOI:** 10.12669/pjms.334.12459

**Published:** 2017

**Authors:** Feng Mingli, Shen Huiliang, Cao Guanglei, Li Zheng, Lu Shibao, Liu Limin, An Shuai

**Affiliations:** 1 Feng Mingli, MD. Department of Orthopaedics, Xuanwu Hospital, Capital Medical University, Changchun Ave 45, Xicheng District, Beijing, 100053, China; 2 Shen Huiliang, MD. Department of Orthopaedics, Xuanwu Hospital, Capital Medical University, Changchun Ave 45, Xicheng District, Beijing, 100053, China; 3 Cao Guanglei, MD. Department of Orthopaedics, Xuanwu Hospital, Capital Medical University, Changchun Ave 45, Xicheng District, Beijing, 100053, China; 4 Li Zheng, MD. Department of Orthopaedics, Xuanwu Hospital, Capital Medical University, Changchun Ave 45, Xicheng District, Beijing, 100053, China; 5 Lu Shibao, MD. Department of Orthopaedics, Xuanwu Hospital, Capital Medical University, Changchun Ave 45, Xicheng District, Beijing, 100053, China; 6 Liu Limin, MD. Department of Orthopaedics, Xuanwu Hospital, Capital Medical University, Changchun Ave 45, Xicheng District, Beijing, 100053, China; 7 An Shuai, MD. Department of Orthopaedics, Xuanwu Hospital, Capital Medical University, Changchun Ave 45, Xicheng District, Beijing, 100053, China

**Keywords:** Arthroplasty, Failed internal fixation, Hip fracture

## Abstract

**Background & Objective:**

Hip fracture is common osteoporotic fracture associated sometimes with failed internal fixation. Joint replacement is commonly used to salvage failed internal fixation of hip fractures (FIFHF). Our objective was to present the outcome of Arthroplasty after FIFHF in our patients.

**Methods:**

A prospective analysis was made on consecutive patients who underwent prosthetic replacements for FIFHF in Orthopaedics Department of Xuanwu Hospital between June 2012 and January 2015. Fifty six patients were included. There were 32 cases of failed internal fixation of femoral neck fracture (FIFFNF) and 24 cases of failed internal fixation of intertrochanteric fracture (FIFIF). The reoperations included 36 cases of total hip replacements, and 20 cases of bipolar femoral head replacements. Cemented prostheses were used in 19 patients (long-stems in 7 patients), and uncemented prostheses in 37 patients (long-stems in 12 patients). The patients were followed up for a minimum of 12 months or until their death.

**Results:**

Two patients died of pulmonary infection in the perioperative period, two died of myocardial infarction in two months after the operation, and the rest survived 12 month follow-up at the least. The mean Harris hip scores of the patients were 47 and 85 before and after the operation, respectively. The rate of Excellent and Good results is 82.7%. T tests showed that difference between pre and post-operation Harris hip scores is statistically significant (p <0.001).

**Conclusions:**

Arthroplasty may serve as a suitable salvage technique for FIFHF, and long-stem prosthesis replacement is proved to obtain reliable curative effect, especially in FIFIF.

## INTRODUCTION

Fractures in elderly patients frequently occur due to osteoporosis. Nearly half of these are hip fracture and the incidence increases with the increasing age.^1^,^2^ Femoral neck fracture and intertrochanteric fracture are the main types of hip fracture, both accounting for 49% of all hip fractures, respectively.^3^ The main treatments for femoral neck fractures include cannulated screw fixation, which is usually used in non-displaced fractures or fractures in young patients, and joint replacement.^4^ Most patients with intertrochanteric fracture are treated with internal fixation, either plate fixation or intramedullary fixation. Now some of the patients with severe osteoporosis or comminuted intertrochanteric fracture are treated with arthroplasty.^5^

Internal fixation of both femoral neck and intertrochanteric fractures may fail, and the reasons for the failure include nonunion, loosening of internal fixation, osteonecrosis, traumatic arthritis, malunion, infection, etc.^6^ Previous research has shown that fixation failure in hip fractures ranges from 5% in peritrochanteric fractures through to 15% and 41% in undisplaced and displaced fractures of the femoral neck, respectively.^7^ Reoperation for failed internal fixation of hip fractures (FIFHF) is a challenge for surgeons because of bone defect, removal of internal fixation, massive bleeding and so on. Now the leading treatment in elderly patients for FIFHF is arthroplasty.^8^

The aim of this study was to present the outcome of arthroplasty after FIFHF in our patients and to review the literature to explore the efficacy and choice of prosthesis and to specify the considerations during the operation.

## METHODS

Form June 2012 to January 2015, the prospective study was made on consecutive patients who underwent arthroplasty because of FIFHF, and surgical operations were finished by the same team of surgeons in orthopaedics department of Xuanwu hospital (we have got ethical committee approval). ***Inclusion criteria:*** (1) FIFHF, including cannulated screw fixation for femoral neck fracture and intramedullary or plate fixation for intertrochanteric fracture; (2) etiology of failure: breakage of internal fixation device, nonunion, traumatic arthritis, displacement of internal fixation device and osteonecrosis; (3) muscle strength of affected side: grade 3 and above; (4) patients with cognitive ability and cooperative in the operation and postoperative rehabilitation; (5) preoperative risk assessment score ≥8.^9^ ***Exclusion criteria:*** (1) patients with progressive malignancy; (2) active rheumatoid; (3) severe anemia; (4) a history of contralateral hip fractures. After inclusion criteria and exclusion criteria, a total of 56 patients, 30 males and 26 females, with a mean age of 72 years (range 56 to 88), were included. The period between the fixation and the replacement was 6-55 months (mean 28 months). There were 32 cases of failed internal fixation of femoral neck fracture (FIFFNF), all done with cannulated screws. 24 patients had failed internal fixation of intertrochanteric fracture (FIFIF), including 7 with PFN, 6 with PFN-A, 2 with LISS plate, and 9 with Asian intramedullary hip screw (Asian IMHS, a type of gamma nail).

The arthroplasty included 36 cases of total hip replacements (THR), and 20 cases of bipolar femoral head replacements. Cemented prostheses were used in 19 patients (long-stems in 7 patients), and uncemented prostheses in 37 patients (long-stems in 13 patients). All the long-stem prostheses were used in patients with nonunion after FIFIF.

### Operation

***(1) Preoperative preparation:*** Venous ultrasonography was performed to identify deep venous thrombosis. The patients were given low molecular weight heparin (LMWH) to prevent thrombosis resulted from lack of movement, which was withdrawn the day before the operation. Disturbances in fluid and electrolytes and anemia were corrected, and complicated diabetes, heart diseases, respiratory diseases or cerebrovascular diseases were treated so as to improve the tolerance.

**Fig. 1:**
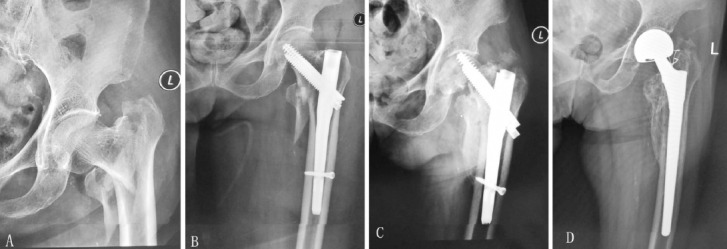
Anteroposterior radiograph of a 73-year-old female showing intertrochanteric fracture treated with intramedullary fixation (A and B). Nonunion result in cut-out of the internal device at one year after operation (C). Anteroposterior radiograph two year after salvage uncemented bipolar long-stem hemiarthroplasty showing good prosthesis position and osteointegration (D).

***(2) Procedures:*** Posterolateral approach to hip was done. Incision was extended towards the distal end of femur as necessary for the removal of fixation devices. For patients with poor physical condition and no evident degeneration or damage of acetabular cartilage, a hemiarthroplasty was conducted. For patients with intertrochanteric fractures, long-stem prostheses (cemented or uncemented depending on bone density and morphology of medullary cavity) were used if it was noted during the surgery that calcar femorale or lesser trochanter fracture was present, and that medial femur could not support the prosthesis sufficiently (Fig. 1). Steel wire or titanium cable fixation was done if nonunion of the greater and/or lesser trochanter with obvious dislocation was noted. Hyperplastic tissues in the joint capsule or the joint cavity were sent to frozen section if infection was suspected. No infection was detected in this study. IV antibiotics were administered half an hour before the operation to prevent infection.

***(3) Postoperative treatment:*** After the surgery, IV antibiotics were given for 24 hours to prevent infection, and subcutaneous injection of LMWH and physiotherapy were performed to prevent thrombosis for 25 days. If deep vein thrombosis (DVT) occurred, a consultation was finished by vascular department to determine treatment plans. The patients were allowed to get up three days after the operation.

***(4) Hip scores and follow-ups:*** Harris hip scores were recorded upon patient admission. Clinic or phone follow-ups were done for a minimum of 12 months or until patients’ death, and the Harris hip scores at 12 months after the operation were recorded for survived patients.

***(5) Statistics:*** T test was used to compare the pre and post-operation Harris hip scores, and p values less than 0.05 was considered statistically significant.

## RESULTS

**Fig. 2:**
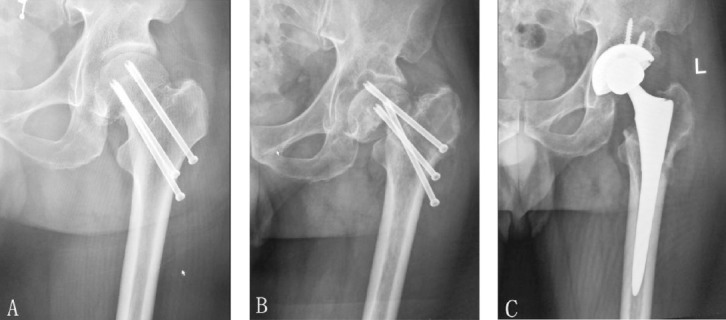
Anteroposterior radiograph of a 68-year-old male showing femoral neck fracture treated with cannulated screws fixation (A). Nonunion result in cut-out of the internal device at one year after operation (B). Anteroposterior radiograph 2 year after salvage uncemented total hip arthroplasty showing good prosthesis position (C).

In this study, two patient died of pulmonary infection in the perioperative period (cemented prosthesis), two died of myocardial infarction two months after the operation (uncemented prosthesis), and the rest survived who were followed-up for a minimum of 12 months. The time of follow-ups was 12-35 months, with a mean of 25 months. No dislocation of prosthesis or infection, or deadly thrombosis occurred, but DVTs occurred in lower legs of 20 patients and were treated successfully with conservative treatment. Harris hip scores of the patients were 32-58 (mean 47) and 68-93 (mean 85) before and after the operation, respectively. There were 14 Excellent, 29 Good, 7 Fair and 2 Poor cases. T tests showed that the difference between pre and post-operation Harris hip scores was statistically significant (p <0.001 F=0.083 t=-30.483). No evidence of loosening or sinking of prosthesis was seen in the follow-up X-ray (Fig. 2). There was no osteolysis at the uncemented prosthesis-bone interface. According to the Barrack grading system,^10^ the quality of cement was graded A in 9 cases, and B in 8 cases.

## DISCUSSION

In this study, the mean Harris hip scores of the patients were 47 and 85 before and after the operation, respectively, and the rate of Excellent and Good results is 82.7%. Although arthroplasty is commonly used to salvage FIFHF and satisfactory curative effect can been achieved by arthroplasty, orthopaedic surgeons still face with challenges. This part mainly discusses reasons for FIFHF, how to avoid it, to explore the efficacy and choice of prosthesis in arthroplasty for FIFHF, and to specify the considerations during the operation.

### 1. High frequency of and reason for FIFHF

Femoral neck fracture and intertrochanteric fracture are the main types of hip fractures,^6^ and 90% of the patients are 60 years or older.^11^ Hip fractures often result in disability or death,^12^ which can be reduced by surgical treatments.^13^ Patients with femoral neck fractures are treated mainly with cannulated screw fixation, which is usually used in non-displaced fractures or fractures in young patients, or arthroplasty;^4^ most patients with intertrochanteric fracture undergo internal fixation, either plate fixation or intramedullary fixation. It was reported that 96%-98% of intertrochanteric fractures were treated with internal fixation in America over the last decade; in patients with femoral neck fractures, two-thirds of patients under the age of 60 underwent internal fixation while two-thirds of patients aged 60 or above underwent joint replacement.^14^ Internal fixation of both femoral neck and intertrochanteric fractures may fail. Previous research has shown that fixation failure in hip fractures ranges from 5% in peritrochanteric fractures through to 15% and 41% in undisplaced and displaced fractures of the femoral neck, respectively.^14^ Examples of failed cases included nonunion, loosening of internal fixation, osteonecrosis, traumatic arthritis, malunion, infection, etc.^6^ In this study, there were 32 patients with femoral neck fracture, of whom 23 had displaced fracture. Displaced femoral neck fracture significantly increased the rate of nonunion and osteonecrosis.

Murphy et al’s study showed that the reoperation rate for nondisplaced fractures treated with fixation was 15% and for displaced fractures 38% after fixation and 7% after hemiarthroplasty; internal fixation and displacement were associated with increased reoperation rates; hemiarthroplasty resulted in fewer reoperations than internal fixation.^15^ Tidermark et al.^16^ studied 102 patients with displaced fracture of the femoral neck who treated either by open reduction and internal fixation (ORIF) or THR. The rate of revision procedure was 4% in the THR group and 42% in the ORIF group; THR group was better in reviews regarding movement, pain and quality of life. Osteoporosis can also lead to failed internal fixation. Heetveld et al.^17^ reported that patients with declined bone mineral density and osteoporosis had a significantly increased rate of reoperation after internal fixation. Reduction of fracture and operating time are also risk factors for failed internal fixation.

A study by Hoelsbrekken et al.^18^ showed that anatomical reduction of displaced femoral neck fractures and performing surgery within 48 hours was associated with reduced rate of failure of internal fixation. In a prospective study by Clement et al.^19^, it was concluded that posterior tilt of the femoral neck is an independent predictor of fixation failure in patients with Garden I fracture. Internal fixation of intertrochanteric fractures, which has a failure rate of approximately 5%,^7^ has a higher success rate than fixation of femoral neck fractures. The failure rate will increase in comminuted or unstable fractures. Karthik K’s study shows that failure of internal fixation of unstable intertrochanteric fractures often occurs in patients with osteoporosis.^20^

### 2. How to choose the right treatment for primary hip fracture

The main treatments for primary femoral neck fracture include cannulated screw fixation and arthroplasty, and the former is generally considered a treatment for non-displaced fractures or fractures in young patients.^4^ For patients of 60 years or older with Garden I & II fractures complicated by severe osteoporosis and other severe internal medicine diseases, one-stage arthroplasty should be used because of their poor tolerance for reoperation. A study on 162 elderly (≥65 years old) patients with Garden stage I or II femoral neck fractures showed that the grading of American Society of Anesthesiologists was correlated with fixation failure, and that patients with poor physical condition tended to have higher failure rate of internal fixation.^9^ A study found that compared with internal fixation, arthroplasty may result in a lower rate of subsequent reoperation at mid- and long-term followup, and better mid-term functional recovery.^14^ In addition, arthroplasty allows patients to get up and begin rehabilitation early, and reduces the complications and rate of disability and death.^7^

Intertrochanteric fractures are usually treated with internal fixation. The rate of FIFIF is lower than that for femoral neck fractures, but reoperation for FIFIF is more difficult. Therefore, some of the patients with severe osteoporosis or comminuted intertrochanteric fractures are treated with arthroplasty. Karthik’s study suggested that hemiarthroplasty is a reliable alternative to internal fixation in elderly osteoporotic patients with unstable intertrochanteric fractures.^20^ Mäkinen et al suggested that arthroplasty for unstable pertrochanteric fractures has therefore been considered as an alternative primary treatment option.^21^ Lee et al’s study showed that cementless bipolar hemiarthroplasty using an extensively hydroxyapatite-coated long stems is a useful option for the treatment of unstable intertrochanteric fracture in senile patients with severe osteoporosis.^22^

### 3. Revision procedures for FIFHF and considerations during the operation

Internal fixation is an important treatment for hip fracture, though the failure of it cannot be avoided. Reoperation is required for most of the patients who have FIFHF, and revision procedures include refixation, osteotomy, arthroplasty, etc. Method of revision is decided based on the severity of hip deformity physical condition, presence of osteoporosis, status of acetabular cartilage and presence of femoral head ischemia, etc. Refixation is used for most of the young patients who have FIFIF if infection is not present, and has a quite high success rate.^23^ However, in older patients who have FIFHF, refixation may suffer failure.. Stepanovic et al reported that a case of 68-year-old female patient suffered FIFIF (sliding hip screw). Revision surgery of intertrochanteric fracture nonunion was performed with short Gamma nail and bone grafting. However, the nail failure occurred again after implantation. They concluded that this undesired series of events were caused by inappropriate implant selection, thus replacing the nail to a modular THA was more effective as salvage surgery.^24^ A case of our study, 65-year-old female patient suffered refixation failure (plate and screws) following trochanteric fixation failure revision surgery (intramedullary nail). Then a cemented hemiarthroplasty was used and the patient got better mid-term functional recovery.

In this study, hemiarthroplasty was adopted for aged patients in poor condition who had intact acetabular cartilage. A study demonstrated no statistically significant difference between hemiarthroplasty and THA for femoral neck fractures in mid-term revision surgery.^25^ Uncemented prosthesis was our first choice because it can avoid cement extrusion from screw holes and the influence of cement on hemodynamics. However, for some patients, the uncemented prosthesis cannot fit into the medullary cavity, for example, “stovepipe” type femoral medullary cavity, thus a cemented prosthesis is used. In this study, long-stem prostheses were used in the revision surgeries for 19 patients who had FIFIF, and the outcomes were satisfactory. During the operation for these patients, bone defects on top of the lesser trochanter, and deficiency of prosthesis in proximal weight-bearing could be noted. A long-stem prosthesis allows the distal medullary cavity to bear weight, and bridges the holes for internal fixation.^8^ Dislocation of greater and lesser trochanter due to fracture is reduced and fixed with steel wire or cable to restore the muscle attachments.

Anders Enocson et al’s study suggested that standard-length femoral stems had an increased risk of reoperation compared to long stems, and that long femoral stems should bridge previous holes and defects to reduce the risk for reoperation after failure of fixation of a intertrochanteric or subtrochanteric fracture.^26^ The procedure of arthroplasty after FIFFNF is similar to that of the primary replacement, whereas arthroplasty after FIFIF is complex. A comprehensive assessment was performed before the operation, and the fixation device was removed following careful preparation. Some of the cannulated screws, which were difficult to screw out, were removed after detaching femoral head. In this study, removal of device was done this way in four patients. In five patients, it was difficult to remove the intramedullary nails and dislocate the femoral head, so greater trochanter osteotomy was done. The greater trochanter was fixed with steel wire or titanium cable after the placement of prosthesis. Sclerotic bone was developed in the medullary cavity after the internal fixation of intertrochanteric fracture, which should be removed during the reaming so that the prosthesis fit in perfectly. Operators should take note of the extrusion of cement caused by holes made in the internal fixation.

Our study has some limitations. First of all, the study did not included a large sample; Second, patients with FIFHF came from different trauma centers, and internal fixation devices are not exactly the same; Third, the follow-up time, with a mean of 25 months, is not long enough. However, reoperation is to salvage operation, at the same time, the patients is the elderly with a mean of 72 years old. So evaluation of the curative effect should not require more than 2.5 years of follow-up for reoperation.

## CONCLUSIONS

Arthroplasty may serve as a suitable salvage technique for FIFHF, and long-stem prosthesis replacement is proved to obtain reliable curative effect, especially in FIFIF.

### Authors’ Contributions

***Feng Mingli:*** Conception & design and Drafting the article.

***Shen Huiliang and Lu Shibao:*** Analysis & interpretation of data.

***Cao Guanglei:*** Collection of date.

***Li Zheng:*** To analysis and sum up the literatures concerned.

***Liu Limin:*** Revising manuscript.
